# Trimetazidine attenuates pressure overload-induced early cardiac energy dysfunction via regulation of neuropeptide Y system in a rat model of abdominal aortic constriction

**DOI:** 10.1186/s12872-016-0399-8

**Published:** 2016-11-17

**Authors:** Ailan Chen, Wanglin Li, Xinyu Chen, Yuechun Shen, Wenjun Dai, Qi Dong, Xinchun Li, Caiwen Ou, Minsheng Chen

**Affiliations:** 1 Department of Cardiology, The First Affiliated Hospital of Guangzhou Medical University, Guangzhou, 510120 China; 2 Department of Gastrointestinal Surgery, Affiliated Guangzhou First Municipal People’s Hospital, Guangzhou Medical University, Guangzhou, 51018 China; 3 Department of Pathogenic Biology, Guangzhou Hoffmann Institute of Immunology, Guangzhou Medical University, Guangzhou, 511436 China; 4 Department of Cardiology, The Third Affiliated Hospital of Guangzhou Medical University, Guangzhou, 510150 China; 5 Department of Physiology, Department of Medical Experimental Center, Guangzhou Medical University, Guangzhou, 510182 China; 6 Department of Radiology, The First Affiliated Hospital of Guangzhou Medical University, Guangzhou, 510120 China; 7 Department of Cardiology, Zhujiang Hospital of Southern Medical University, Guangzhou, 510280 China

**Keywords:** Metabolic remodeling, Trimetazidine, Ventricular hypertrophy, Neuropeptide Y

## Abstract

**Background:**

Metabolism remodeling has been recognized as an early event following cardiac pressure overload. However, its temporal association with ventricular hypertrophy has not been confirmed. Moreover, whether trimetazidine could favorably affect this process also needs to be determined. The aim of the study was to explore the temporal changes of myocardial metabolism remodeling following pressure-overload induced ventricular hypertrophy and the potential favorable effect of trimetazidine on myocardial metabolism remodeling.

**Methods:**

A rat model of abdominal aortic constriction (AAC)-induced cardiac pressure overload was induced. These rats were grouped as the AAC (no treatment) or TMZ group according to whether oral trimetazidine (TMZ, 40 mg/kg/d, for 5 days) was administered. Changes in cardiac structures were sequentially evaluated via echocardiography. The myocardial ADP/ATP ratio was determined to reflect the metabolic status, and changes in serum neuropeptide Y systems were evaluated.

**Results:**

Myocardial metabolic disorder was acutely induced as evidenced by an increased ADP/ATP ratio within 7 days of AAC before the morphological changes in the myocardium, accompanied by up-regulation of serum oxidative stress markers and expression of fetal genes related to hypertrophy. Moreover, the serum NPY and myocardial NPY-1R, 2R, and 5R levels were increased within the acute phase of AAC-induced cardiac pressure overload. Pretreatment with TMZ could partly attenuate myocardial energy metabolic homeostasis, decrease serum levels of oxidative stress markers, attenuate the induction of hypertrophy-related myocardial fetal genes, inhibit the up-regulation of serum NPY levels, and further increase the myocardial expression of NPY receptors.

**Conclusions:**

Cardiac metabolic remodeling is an early change in the myocardium before the presence of typical morphological ventricular remodeling following cardiac pressure overload, and pretreatment with TMZ may at least partly reverse the acute metabolic disturbance, perhaps via regulation of the NPY system.

## Background

Despite significance advances in therapeutic strategies against congestive heart failure (CHF) during the past few decades, CHF remains one of the most serious diseases, contributing to substantial mortality and morbidity throughout the world [1, 2]. Ventricular hypertrophy and cardiac remodeling have been well established as important pathologic events that play fundamental roles during the pathogenesis of CHF [3, 4]. Indeed, various stimuli of cardiac injury lead to impairment of cardiac function and subsequently activation of neurohormonal factors, which in the long-term, causes apoptosis of cardiomyocytes, fibrosis and eventually hypertrophy of the myocardium, finally leading to a vicious cycle of cardiac function deterioration [5]. This pathogenetic process has been known as ventricular remodeling, and inhibitors of neurohormonal factors that are involved in the activation of ventricular remodeling have been recognized as important therapeutic medications against CHF [6]. More importantly, subsequent studies have highlighted the role of myocardial metabolic dysfunction as a potential pivotal process before ventricular remodeling begins during the pathogenesis of CHF [7]. Hypertrophied myocardium has been shown to be characterized by the disturbance of homeostasis of energy metabolism [8–11]. Moreover, an interesting genomic study found that early and acute changes in the expression of genes related to energy metabolism can be detected before evidence of cardiac remodeling [12], which indicates that disturbance of myocardial biogenesis may be an initial process and important contributor to cardiac remodeling. However, to the best of our knowledge, the acute changes in energy status during the early phase of cardiac pressure overload, a conventional stimulus of cardiac remodeling, have not been systematically studied.

Neuropeptide Y (NPY) has been recognized as a key regulator of energy metabolism via its binding and signaling with its receptors (NPY-R) [13]. Previous studies in human patients and animal models of CHF have suggested that NPY may participate in the pathogenesis of myocardial hypertrophy and subsequent CHF [14–16]. However, whether the NPY system is involved in the metabolism disturbance during the early phase of cardiac pressure overload still needs to be determined. Trimetazidine (TMZ) is used clinically as an anti-angina medication, because it can improve energy metabolism during the process of myocardial ischemia [17, 18]. Upon application of TMZ, the utilization of free fatty acids for energy production is decreased in myocardium due to its inhibitory effect on mitochondrial 3-ketoacyl CoA thiolase (3-KAT) in beta-oxidation, and subsequently, the consumption of non-lipid substrates, mainly glucose, is increased [19]. Recent studies suggested that TMZ may inhibit pressure overload-induced cardiac fibrosis and myocardial remodeling [20]. Therefore, it is reasonable to speculate that TMZ, as a medication capable of optimizing myocardial metabolism, may attenuate the disturbance of myocardial energy metabolism during the acute phase of pressure overload-induced myocardial remodeling.

Based on the evidence presented above, we hypothesized that myocardial metabolic disturbance may be an early event before the morphological changes of myocardial hypertrophy, and changes in the NPY system may be involved. Moreover, pretreatment with TMZ may attenuate the metabolic disturbance of the myocardium before the morphological changes of ventricular hypertrophy can be detected, and these metabolic benefits of TMZ may involve the regulation of serum and myocardial NPY system. Therefore, in this study, we applied a rat model of short-term (within 7 days) abdominal aortic constriction (ACC)-related cardiac pressure overload but without significant morphological changes of myocardial hypertrophy, to systematically observe the changes of myocardial energy metabolism, potential benefits of TMZ pretreatment, and the roles of the NPY system involved.

## Methods

The current study was performed in accordance with the Guide for the Care and Use of Laboratory Animals published by the US National Institutes of Health (NIH Publication No. 85-23, revised 1996). The experiments protocols were approved ahead of performance by the Committee on Ethics of Animal Experiments of Guangzhou Medical University.

### Animal models of cardiac pressure overload

Thirty-eight male Wistar rats, weighing 60 ± 10 g, were purchased from the Animal Experimental Center of the Southern Medical University. Rats were given free access to food and water as needed, housed in a stable space with the room temperature at 22 °C, and maintained on a 12-h light/dark cycle for 7 days before the ACC procedure.

These rats were randomized to three groups: (1) the sham group (*n* =12), in which the surgical procedure of ACC was performed in the same way except for ligation of the abdominal aorta; (2) the AAC group (*n* = 13), in which AAC was performed by ligation of the abdominal aorta 5 mm above the branching of the left renal artery to induce pressure overload of the heart, as described previously [21, 22]; and (3) the TMZ group (*n* = 13), in which the oral administration of TMZ (40 mg/kg/d, for 5 days) was performed before the surgical procedure for AAC-induced cardiac pressure overload. Briefly, all surgical procedures were performed after the rats were well anesthetized with intraperitoneal sodium pentobarbital (45 ml/kg). The abdominal aorta, proximal to the left renal artery was exposed and separated from the vena cava. A 2-0 silk suture was tied beside the aorta between the branches of the celiac and the anterior mesenteric arteries, using a blunt 24 G probe with the external diameter of 0.55 mm. After the above procedure, 1 ml saline was administered in the peritoneal cavity of each rat to replenish of the loss of the fluid during the procedure. Then, the abdominal wall was closed and the skin was sutured. The rats in the sham group received the same procedure expect that the silk suture around the aorta was pulled through and not tied. Ampicillin (50 mg/kg, i.m.) was injected once daily for 3 days after surgery to prevent infection, and all rats were placed on a warm pad for recovery after the surgery. Overall, one rat from the AAC group and one rat from the TMZ group died from acute heart failure after the surgical procedure. Twelve rats in each group survived the surgical procedures, and these rats within each group were further divided in to two groups (6 rats in each for the different observation periods of 2 and 7 days).

### Echocardiographic examination

The cardiac systolic function and dimensions of the heart chambers were measured 2 and 7 days after surgery, according to the different observation groups, by two-dimensional echocardiography (Philips IE 33,) by an experienced investigator who was blinded to the treatment groups. Measurements were recorded as the mean of at least three consecutive cardiac cycles. The parameters recorded included interventricular septal thickness at end diastole (IVSd), left ventricular posterior wall thickness at end diastole (LVPWd), left ventricular end-diastolic dimension (LVEDD), left ventricular end-systolic dimension (LVESD), left ventricular factional shortening (LVFS), and left ventricular ejection fraction (LVEF).

### Blood sampling and measurements of serum levels of NPY, MDA, and SOD activity

After echocardiologic examination, blood samples of rats from each group were obtained via intubation of the carotid artery. Immediately, blood samples were centrifuged at 12,000 g for 10 min at 4 °C, and then the supernatant was collected for measurement at −20 °C. Serum levels of NPY were measured using a commercially available enzyme immunoassay (EIA) kit (Bachem Laboratories, S-1145), and the experimental procedures were performed according to the manufacturers’ procedures. Similarly, the serum level of malonaldehyde (MDA) and serum superoxide dismutase (SOD) activity were evaluated using a suite of commercial kits, in accordance with the manufacturer’s instructions (Beyotime Institute of Biotechnology, Nanjing, China).

### Determining the ratio of heart to body weight

After blood sampling, the rats were sacrificed, and hearts from rats in each group were excised. The heart weight (HW) was obtained as well as the weight of the left ventricle (LVW). Subsequently, the ratio of HW to body weight (BW) (HW/BW) was calculated. After measurement, the left ventricle sample was excised, rinsed with ice-cold saline, and dried with blotting paper. Next, the myocardial sample was placed in a microtube, flash frozen in liquid nitrogen, and finally stored at −80 °C for use in subsequent molecular biological experiments.

### Measurement of cell surface area

Some myocardial tissue samples were fixed in 4% formaldehyde and embedded in paraffin. These tissues were then sectioned at 5 μm and stained with hematoxylin and eosin (HE). The cell surface area (CAS) of cardiomyocytes was then measured and analyzed with IPP6.0 Software (Leica, Germany). At least 100 cells were selected for analysis from each section of the myocardial tissue.

### ADP/ATP ratio assay

To assess the energy status of the myocardial tissue, an ADP/ATP ratio assay was performed using the ApoSENSOR™ ADP/ATP Ratio Assay Kit (MBL, Nagoya, Japan) according to the manufacturer’s instructions.

### Quantitative real-time PCR

Total RNA from myocardial tissue samples of rats in each group were extracted using Trizol reagent (Invitrogen Life Technologies, Thermo Fisher Scientific) and reverse transcribed into cDNA (Advantage RT-for-PCR kit; Clontech, Palo Alto, CA, USA) for subsequent analysis of the mRNA levels of myocardial hypertrophy-related genes including atrial natriuretic factor (ANF) and β myosin heavy chain (MHC-β), as well as NPY-1R, 2R, and 5R. Briefly, 1 μg of total RNA was reverse transcribed using 2 μg of random primers and Moloney murine leukemia virus reverse transcriptase according to the manufacturer’s protocol (Advantage RT-for-PCR kit). We used a One-Step system (Applied Biosystems, Thermo Fisher Scientific) to perform the real-time PCR analysis. The sequences of the primers for the genes analyzed are listed in Table 1. We applied the relative quantitation method to measure the expression levels of the genes of interest and analyzed the results of two independent experiments with triplicate sampling. For each experimental sample, a gene was considered as not expressed if amplification was not detected by threshold cycle Ct = 40. The results were expressed in arbitrary units of ΔCt, which represented targeted mRNA transcripts.

**Table 1:** Sequences of primers used for real-time RT-PCR analysis

NPY-1R-F	5′GCTGTGGAACGTCATCAGCTA3′
NPY-1R-R	5′TTGATAGATCACGAAGGGCAG3′
NPY-2R-F	5′CCCGGATCTGGAGTAAGCTAAA3′
NPY-2R-R	5′GTGGAGCACATCGCAATAATGT3′
NPY-5R-F	5′CAATACAGCTGCTGCTCGGA3′
NPY-5R-R	5′AAATCGTCTACGCTGCCTCTG3′
ANF-F	5′GGGGGTAGGATTGACAGGAT3′
ANF-R	5′CTCCAGGAGGGTATTCACCA3′
β-MHC-F	5′CCTCGCAATATCAAGGGAAA3′
β-MHC-R	5′TACAGGTGCATCAGCTCCAG3′
β-actin-F	5′GACAGGATGCAGAAGGAGATTACT3′
β-actin-R	5′TGATCCACATCTGCTGGAAGGT3′

*F* forward, *R* reverse

### Western blot analyses

Total proteins in myocardial tissue samples were ground and homogenized with a protein lysis solution (KeyGen BioTech, China). Briefly, myocardium form the LV was frozen in liquid nitrogen and then homogenized in phosphate-buffered saline (PBS) with a protease inhibitor cocktail (Roche, South San Francisco, CA, USA) using a tissue grinder. Homogenates were centrifuged at 14,000 rpm for 15 min at 4 °C. Nuclear and cytoplasmic proteins were separated according to the protocol described in the Nuclear and Cytoplasmic Extraction Kit (Thermo Scientific, USA). Supernatants were collected and assayed for total protein using the BCA method (KeyGen BioTech, China). Equal amounts of protein (125 μg) were resolved on 10% Tris-glycine sodium dodecyl sulphate (SDS) polyacrylamide gels. Protein bands were blotted onto nitrocellulose membranes. After blocking in 5% dried milk in Tris-buffered saline containing Tween-20 for 1 h at room temperature, membranes were incubated for 24 h at 4 °C with one of the following antibodies: anti-NPY-1R polyclonal antibody (Abnova, Taiwan, China), anti-NPY-2R (D42) pAb (Bioworld, USA), anti-NPY-5R (M264) pAb (Bioworld), and anti GAPDH (14C10) Rabbit mAb (Cell Signaling Technology, USA) to detect the proteins levels. Membranes were incubated for 1 h at room temperature with horseradish peroxidase-conjugated donkey anti-rabbit immunoglobulin (1:10,000; Santa Cruz Biotechnology). Peroxidase labeling was detected using the enhanced chemiluminescence Western blotting detection system (Amersham Pharmacia Biotech, Piscataway, NJ, USA) and analyzed by densitometry. The optical density values were normalized to that of GAPDH.

### Statistical analyses

Statistical analyses were performed using SPSS 11.0 (SPSS Inc., Chicago, IL, USA). The results are reported as mean ± standard deviation (SD). Differences were analyzed for significance by one-way or two-way analysis of variance (ANOVA), and *p* < 0.05 was considered to be statistically significant.

## Results

### Effects of TMZ on left ventricular hypertrophy and cardiac function in AAC rats

**Fig. 1:**
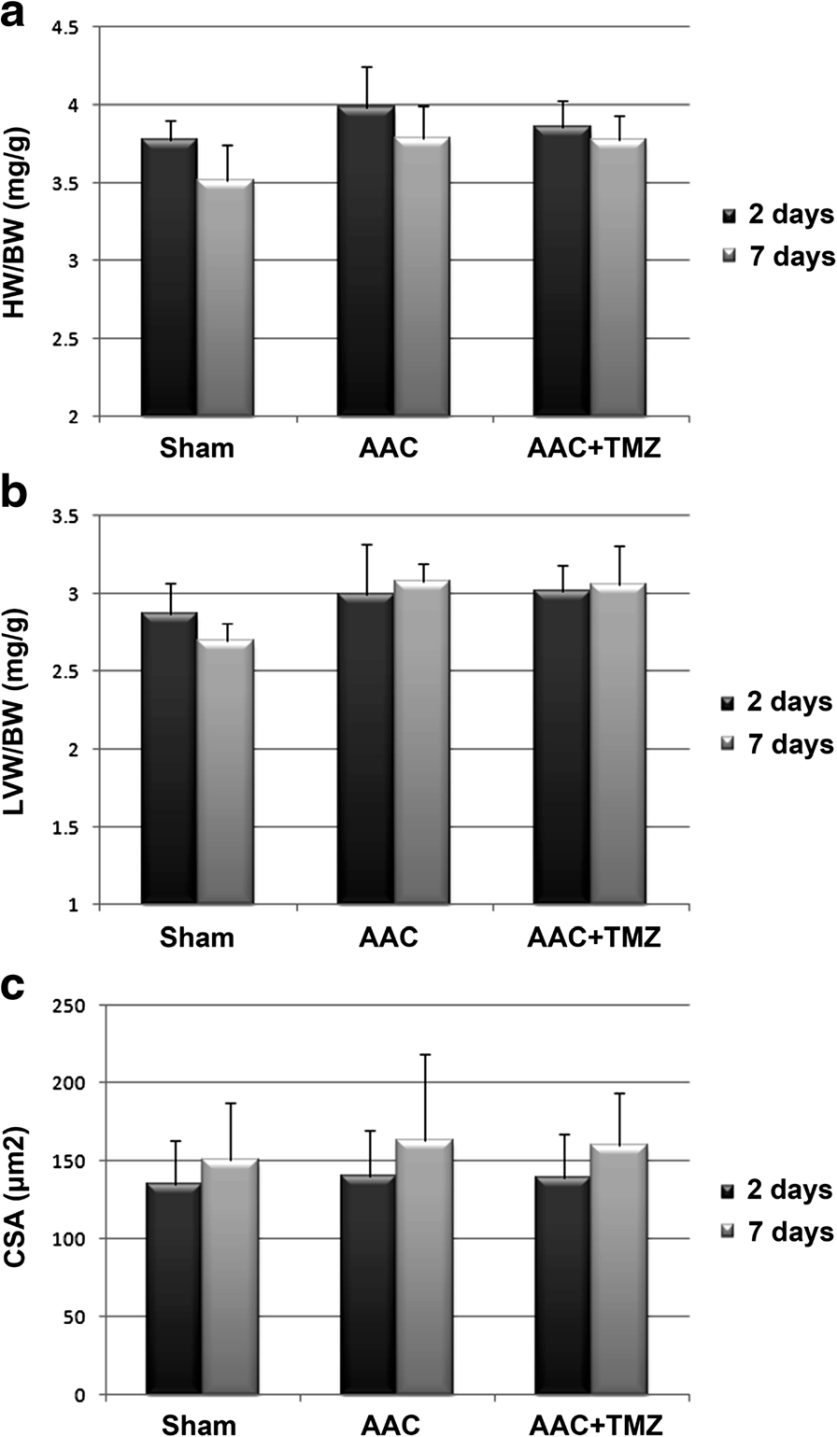
Comparisons of the heart weight, left ventricular weight, and cell surface area of cardiomyocytes for rats from each group. **a**, Effects of AAC and TMZ pretreatment on HW/BW in rats from each group; **b**, effects of AAC and TMZ pretreatment on LVW/BW in rats from each group; **c**, effects of AAC and TMZ pretreatment on CSA of cardiomyocytes for rats from each group. *AAC* abdominal aortic constriction. *TMZ* trimetazidine, *HW* heart weight, *BW* body weight, *LVW* left ventricular weight, *CSA* cell surface area; *n* = 6 per group; 10 high magnification views with at least 100 cardiomyocytes in each view were analyzed to determine the CSA

To observe the acute impact of pressure overload on cardiac hypertrophy and cardiac function, we measured the dimensions of the left ventricle and cardiac systolic function via echocardiography. The results of the echocardiographic examination showed that the parameters of left ventricular dimensions, such as IVSd, LVPWd, LVEDD, and LVESD, as well as the indices of cardiac systolic function, including LVFS and LVEF, were not significantly different between the rats from the sham and AAC groups at 2 or 7 days after AAC (Table 2). Moreover, the results of the gross and cellular pathologic studies revealed that the ratios of HW/BW and LVW/BW and the CSA of the cardiomyocytes of rats in the sham and AAC groups also were not significantly different at 2 or 7 days after AAC (Fig. 1a-c). These results indicate that acute pressure overload did not cause significant changes in cardiac structure or cellular morphology, suggesting that myocardial hypertrophy or cardiac systolic dysfunction was not induced within 7 days of AAC. Moreover, pretreatment with TMZ was not associated with significant changes in the echocardiologically detected cardiac structure or systolic function or the ratios of HW/BW and LVW/BW and the CSA of the cardiomyocytes (Table 1, Fig. 1a-c). These results further indicate that pretreatment with TMZ did not affect the cardiac structure or cellular morphology during the acute phase of AAC (up to 7 days).

**Table 2:** Echocardiologic evaluation of cardiac function at 2 and 7 days after AAC

	2 days after AAC				7 days after AAC			
	Sham	AAC	AAC + TMZ	*P* values	Sham	AAC	AAC + TMZ	*P* values
IVSd (mm)	0.95 ± 0.12	1.00 ± 0.05	1.01 ± 0.06	>0.05	1.10 ± 0.03	1.21 ± 0.07	1.24 ± 0.11	>0.05
LVPWd (mm)	1.30 ± 0.40	1.63 ± 0.07	1.64 ± 0.04	>0.05	1.45 ± 0.19	1.67 ± 0.12	1.65 ± 0.23	>0.05
LVEDD (mm)	4.30 ± 0.27	4.53 ± 0.39	4.52 ± 0.26	>0.05	4.50 ± 0.28	4.90 ± 0.09	5.03 ± 0.12	>0.05
LVESD (mm)	2.46 ± 0.11	2.52 ± 0.09	2.51 ± 0.07	>0.05	2.49 ± 0.07	2.62 ± 0.24	2.64 ± 0.18	>0.05
LVFS (%)	37.97 ± 9.05	44.66 ± 10.04	45.27 ± 8.13	>0.05	38.63 ± 8.15	44.56 ± 1.89	43.68 ± 2.78	>0.05
LVEF (%)	73.47 ± 10.51	85.40 ± 1.91	84.23 ± 2.32	>0.05	79.5 ± 3.41	86.09 ± 3.19	84.48 ± 2.67	>0.05

Data are presented as mean ± SD, *n* = 6 per group

*AAC* aortic artery constriction, *IVSd* interventricular septal thickness at end diastole, *LVPWd* left ventricular posterior wall thickness at end diastole, *LVEDD* left ventricular end-diastolic dimension, *LVESD* left ventricular end-systolic dimension, *LVFS* left ventricular factional shortening, *LVEF* left ventricular ejection fraction, *TMZ* trimetazidine

### Effects of TMZ on expression of genes related to myocardial hypertrophy in AAC rats

**Fig. 2:**
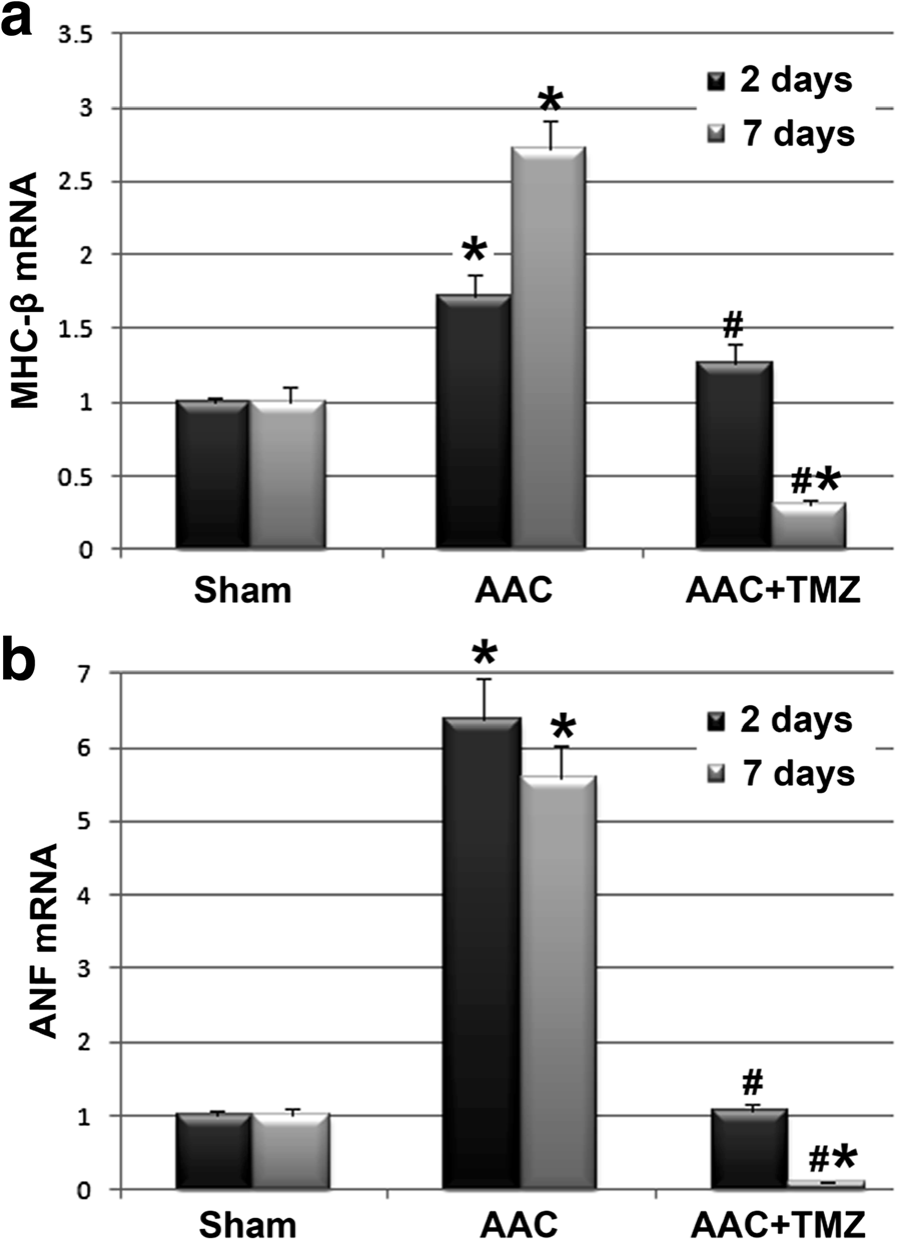
Comparisons of myocardial expression of fetal genes related to myocardial hypertrophy induced by AAC: results of real-time PCR analyses. **a**, Effects of AAC and TMZ pretreatment on mRNA levels of MHC-β in myocardium of rats from each group; **b**, effects of AAC and TMZ pretreatment on mRNA levels of ANF in myocardium of rats from each group. *AAC* abdominal aortic constriction, *TMZ* trimetazidine, *ANF* atrial natriuretic factor, *MHC-β* β myosin heavy chain; *n* = 4~6 per group, and samples were analyzed in triplicate. *, *p* < 0.05 compared with sham group at the same time point; #, *p* < 0.05 compared with AAC group at the same time point

Up-regulated expression of fetal genes, such as ANF and MHC-β, has been recognized as an important feature of the pathogenesis of myocardial hypertrophy [23]. Therefore, we evaluated the acute effects of AAC on the expression of these hypertrophy-related genes, and more importantly, the influence of pretreatment with TMZ on expression of these genes. The results of real-time PCR indicated that the mRNA levels of ANF and MHC-β were significantly increased in the myocardium of rats from the AAC group in comparison with those from the sham group both at 2 and 7 days after AAC (*p* < 0.05, Fig. 2a-b). Moreover, we also found that pretreatment with TMZ inhibited the upregulation of these genes induced by acute AAC as indicated by significantly reduced levels of expression of these genes in the myocardium of rats in the TMZ group as compared with those of the AAC group (*p* < 0.05, Fig. 2). These results suggest that pretreatment with TMZ may inhibit the early upregulation of fetal genes related to myocardial hypertrophy following the acute phase of cardiac pressure overload.

### Effects of TMZ on ratio of ADP/ATP and serum activities of MDA and SOD

**Fig. 3:**
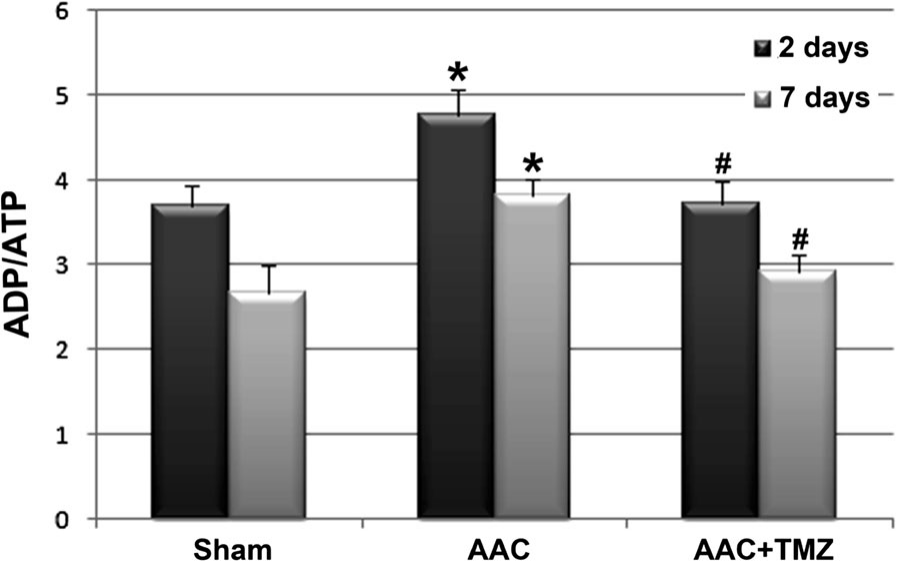
Comparison of ADP/ATP ratios in myocardium. *AAC* abdominal aortic constriction, *TMZ* trimetazidine; *n* = 4~6 per group and samples were analyzed in triplicate. *, *p* < 0.05 compared with sham group at the same time point; #, *p* < 0.05 compared with AAC group at the same time point

**Fig. 4:**
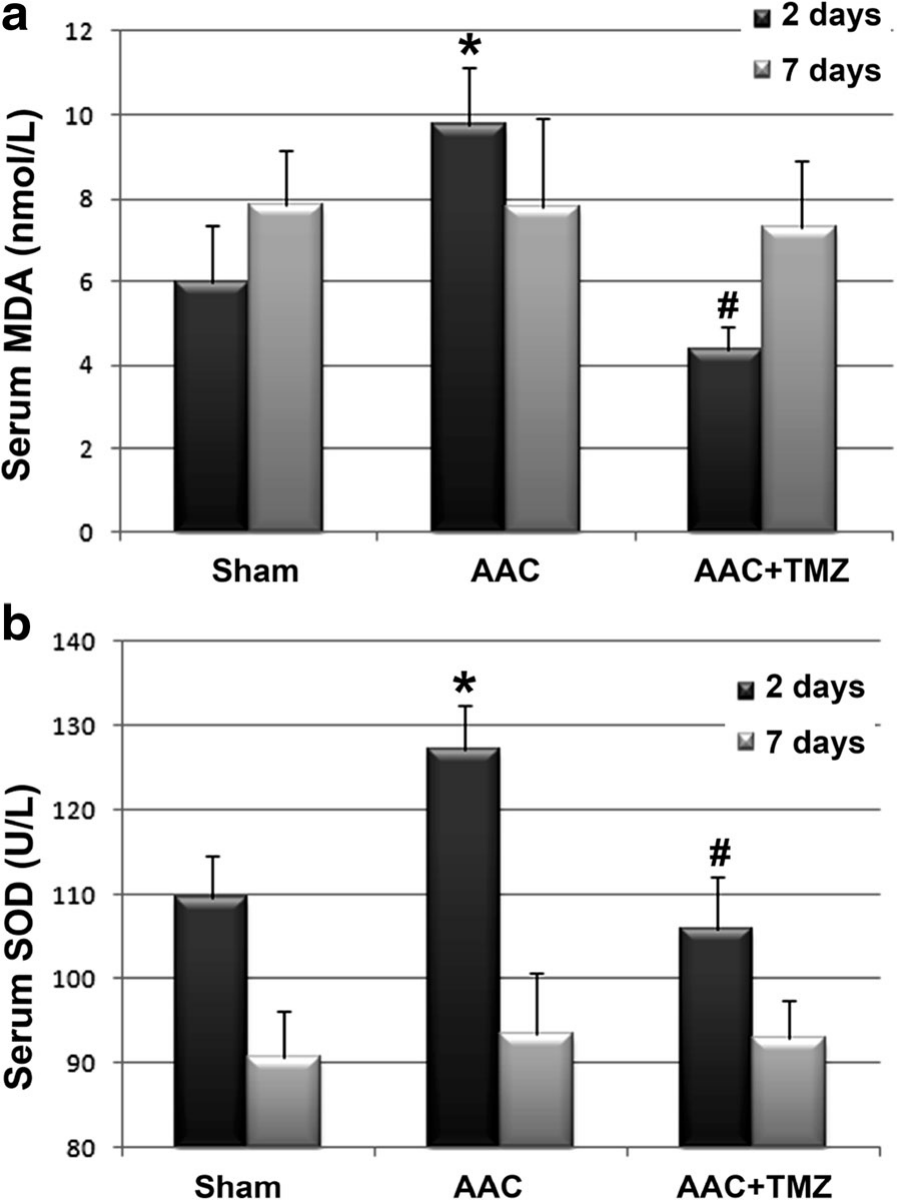
Comparisons of serum activities of oxidative stress markers. **a**, Effects of AAC and TMZ pretreatment on MDA concentrations in rats from each group; **b**, effects of AAC and TMZ pretreatment on SOD activities in rats from each group. *AAC* abdominal aortic constriction, *TMZ* trimetazidine, *MDA* malonaldehyde, *SOD* superoxide dismutase; *n* = 4~6 per group and samples were analyzed in triplicate. *, *p* < 0.05 compared with sham group at the same time point; #, *p* < 0.05 compared with AAC group at the same time point

Previous studies confirmed that the ratio of ADP/ATP is an index of energy metabolism in the myocardium, and disturbance of cardiac energy biogenesis is often associated with an increased ADP/ATP [24]. In this study, we found that acute cardiac pressure overload increased the myocardial ADP/ATP at 2 and 7 days after AAC (*p* < 0.05, Fig. 3). Notably, these changes in metabolic dysfunction seemed to appear before the significant and typical changes in cardiac structure and morphology seen during the late process of AAC. Interestingly, pretreatment with TMZ significantly reduced the myocardial ADP/ATP following AAC (*p* < 0.05, Fig. 3), suggesting that TMZ may favorably affect the energy metabolism of the myocardium even in the acute phase of cardiac pressure overload. Similarly, the significantly increased serum levels of MDA and SOD, which have been considered markers of oxidative stress-related injury detected during the process of energy metabolic dysfunction [25], also were significantly lowered at 2 days after AAC in the rats pretreated with TMZ (*p* < 0.05, Fig. 4a-b). These results indicate that cardiac pressure overload may induce energy metabolic disorder and oxidative stress-related injury before the typical changes of myocardial hypertrophy could be observed, and pretreatment with TMZ may attenuate such disorder of energy biogenesis during the acute phase of AAC.

### Effects of TMZ on the serum NPY levels and myocardial expression of NPY receptors

**Fig. 5:**
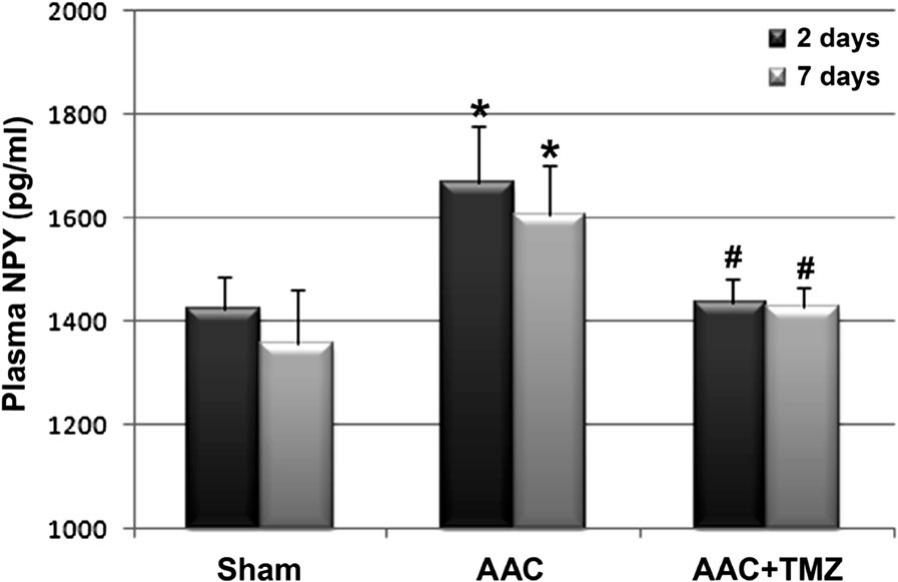
Comparison of serum concentrations of NPY in rats from each group. *AAC* abdominal aortic constriction, *TMZ* trimetazidine, *NPY* neuropeptide Y; *n* = 4~6 per group and samples were analyzed in triplicate. *, *p* < 0.05 compared with sham group at the same time point; #, *p* < 0.05 compared with AAC group at the same time point

**Fig. 6:**
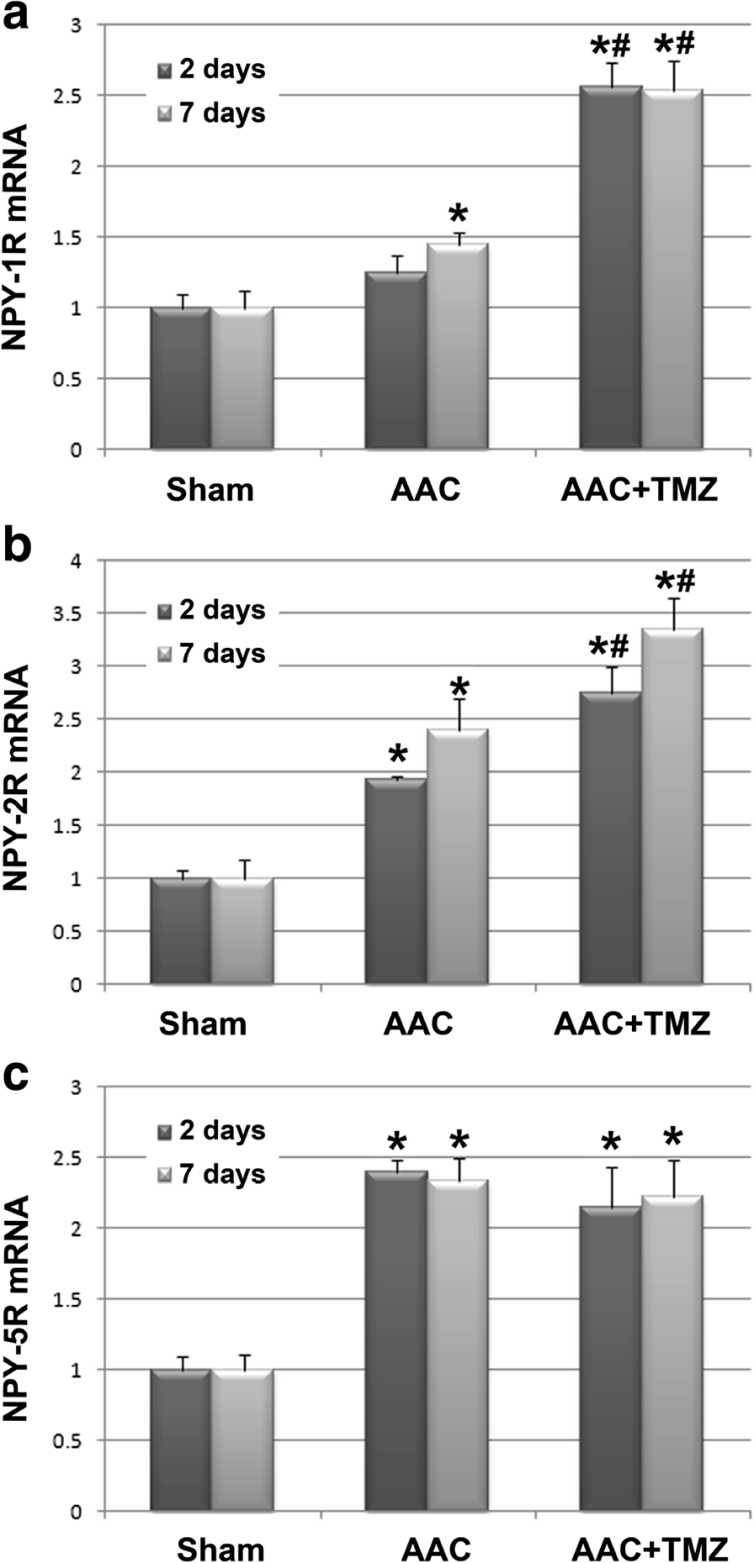
Comparisons of mRNA levels of NPY-1R (**a**), NPY-2R (**b**), and NPY-5R (**c**) in the myocardium of rats from each group: results of real-time PCR analyses. *TMZ* trimetazidine, *NPY* neuropeptide Y; *n* = 4~6 per group and samples were analyzed in triplicate. *, *p* < 0.05 compared with sham group at the same time point; #, *p* < 0.05 compared with AAC group at the same time point

**Fig. 7:**
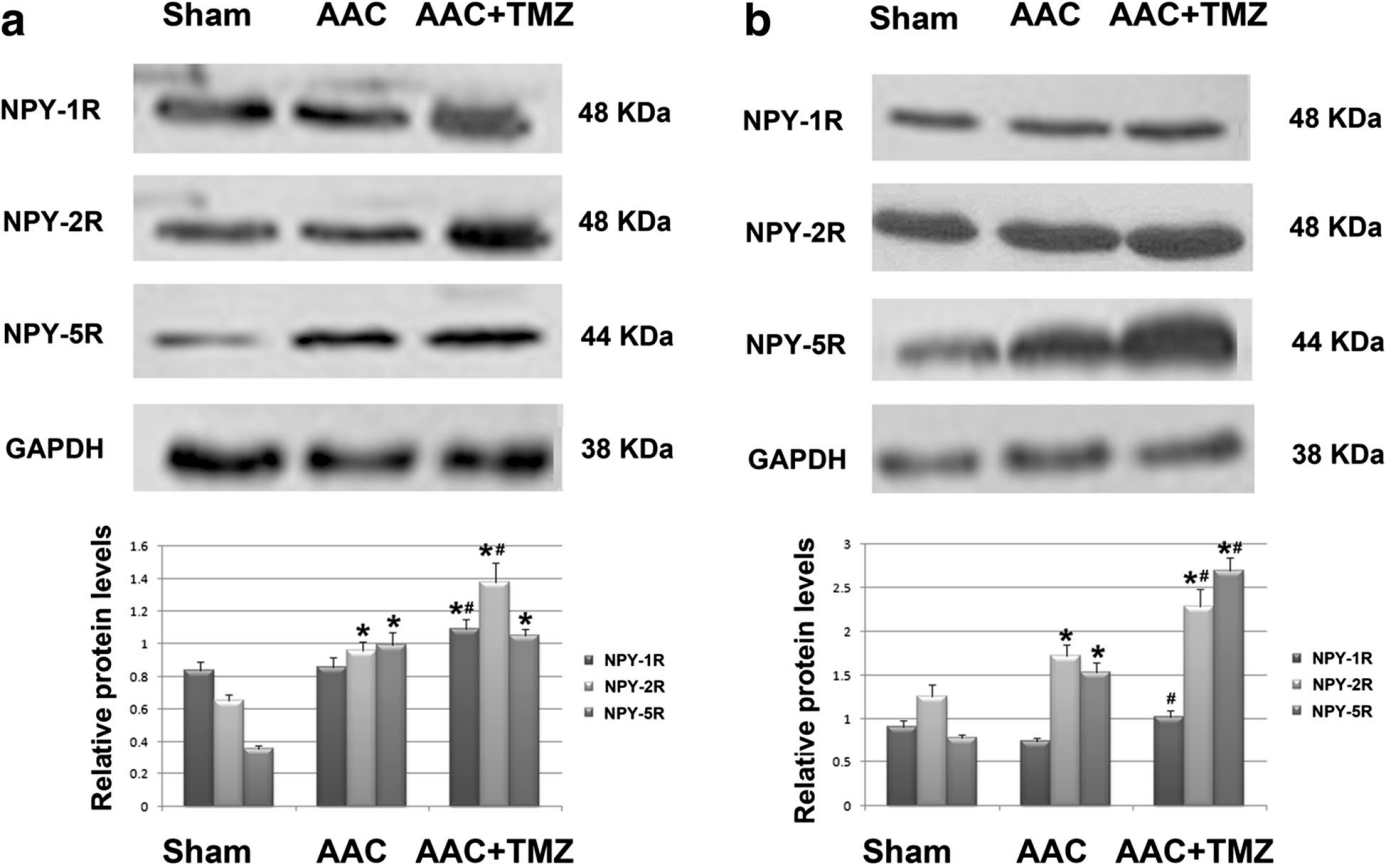
Comparisons of protein levels of NPY-1R, NPY-2R, and NPY-5R in the myocardium of rats from each group at 2 days (**a**) and 7 days (**b**) after AAC: results of western blot analyses. *TMZ* trimetazidine, *NPY* neuropeptide Y, *n* = 4~6 per group and samples were analyzed in triplicate. *, *p* < 0.05 compared with sham group at the same time point; #, *p* < 0.05 compared with AAC group at the same time point

The results of early studies indicated that NPY is an important neurohormonal factor that may function as a key regulator of energy metabolism [13]. In view of the fact that pretreatment with TMZ may attenuate of the disturbance of energy metabolism during the acute phase of AAC, we further evaluated the role of NPY and its receptors during the pathogenesis of cardiac pressure overload, as well as the influence of TMZ pretreatment on them. We found that plasma NPY levels were significantly increased at 2 and 7 days after AAC as compared with those in the sham group (*p* < 0.05, Fig. 5), whereas pretreatment with TMZ significantly inhibited the upregulation of serum NPY during the acute phase of AAC (*p* < 0.05, Fig. 5), suggesting that the favorable effect of TMZ on myocardial energy biogenesis may be related to its inhibitory effect on serum NPY. Moreover, the mRNA levels of myocardial NPY-1R, 2R, and 5R were increased during the acute phase of cardiac pressure overload (*p* < 0.05, Fig. 6a-c), and the mRNA levels of NPY-1R and 2R were further up-regulated following TMZ pretreatment (*p* < 0.05, Fig. 6a-c). However, the mRNA level of NPY-5R was not significantly changed upon TMZ pretreatment as compared with that from the AAC group (*p* > 0.05, Fig. 6c). The results of western blot analyses seemed to parallel those of the mRNA expression analyses. Protein levels of NPY-2R and 5R were increased at 2 days after AAC, and the protein levels of NPY-1R and 2R were further up-regulated following pretreatment with TMZ at 2 days after AAC (Fig. 7a). By 7 days after AAC, protein levels of myocardial NPY-2R and 5R were upregulated, whereas protein levels of all three NPY receptors were further up-regulated after TMZ pretreatment (Fig. 7b). These results suggest that acute cardiac pressure overload may have contributed to early energy dysfunction by inducing the neurohormonal factor NPY and up-regulating NPY-2R and 5R, whereas pretreatment with TMZ attenuated the induction of NPY but further stimulated the protein levels of myocardial NPY receptors.

## Discussion

Our study in a rat model of AAC-induced cardiac pressure overload revealed that myocardial energy metabolic disorder was acutely induced within 1 week after AAC, which was before the typical morphologic changes of ventricular hypertrophy occurred. Moreover, disturbance of myocardial energy metabolism was accompanied by up-regulation of serum levels of oxidative stress markers and expression of myocardial fetal genes related to ventricular hypertrophy. Interestingly, the serum level of NPY and myocardial mRNA and protein levels of NPY-1R, 2R, and 5R also were increased within the acute phase of AAC-induced cardiac pressure overload. More importantly, pretreatment with TMZ could partly attenuate myocardial energy metabolic disturbance, decrease serum levels of oxidative stress markers, attenuate the induction of hypertrophy-related myocardial fetal genes, inhibit the up-regulation of serum NPY levels, and further increase the myocardial expression of NPY receptors. These results confirmed previous findings that energy metabolic disorders may occur prior to typical morphological changes of ventricular hypertrophy following cardiac pressure overload, suggesting a potential pathophysiologic process of metabolic remodeling before myocardial remodeling begins. Moreover, our results for the first time suggest that pretreatment with TMZ, a medication known for its benefits in cardiac energy metabolism, may attenuate the early energy metabolic disturbance of the myocardium during the acute phase of AAC-induced cardiac pressure overload. With respect to the mechanisms underlying the energy remodeling following AAC, we found that changes in serum NPY and myocardial NPY receptor levels may be involved, and TMZ may exert its beneficial effect via regulation of the NPY system. These results highlight the importance of cardiac metabolic remodeling as an early change in the myocardium before the presence of typical morphological ventricular remodeling and suggest that early treatment with TMZ may attenuate the disturbance.

Early studies revealed that disturbance of myocardial energy metabolism, which typically presents as an increased ratio of cellular ADP/ATP, plays important roles in the pathogenesis of CHF [7, 9]. Although this has been proposed in early observations, whether metabolism remodeling occurs before or after the structural changes of myocardial hypertrophy has not been determined until recent years. A genomic study of pressure overload-induced cardiac hypertrophy in mice found early changes in the expression of metabolic genes before the hypertrophic phase of the myocardium [12]. A later study with the same animal model using the quantitative positron emission tomography (PET) imaging also indicated that metabolic maladaptation precedes the onset of severe contractile dysfunction [26]. A recent study in patients with hypertension provided more direct evidence supporting that metabolic remodeling is an early event that occurs before the onset of cardiac hypertrophy. With PET imaging, the investigators found that glucose metabolic remodeling is detectable in hypertensive patients before the development of ventricular hypertrophy [26]. Our study, using an acute model of AAC-induced cardiac pressure overload generated before the presence of typical morphologic changes of ventricular hypertrophy (usually occurring at least 2 weeks after AAC), found that the ADP/ATP was significantly increased, suggesting an occurrence of energy metabolic disturbance before myocardial hypertrophy. Further studies are needed to determine whether strategies targeting early metabolic remodeling could prevent subsequent myocardial structural remodeling.

In view of the fact that TMZ may function to optimize myocardial energy metabolism, we further evaluated whether pretreatment with TMZ could attenuate the metabolism dysfunction during the acute phase of AAC. We found that TMZ not only significantly reversed the changes in myocardial ADP/ATP, but also reduced the serum levels of oxidative stress markers and the myocardial expression of up-regulated fetal genes related to hypertrophy. These results suggest that TMZ may effectively attenuate the metabolic disorder and therefore subsequently inhibit myocardial hypertrophy. Our results are supported by the findings of Liu et al., which showed that TMZ can inhibit myocardial fibrosis, a characterized feature of ventricular remodeling [6], as well as the fact that application of TMZ in patients with CHF is associated with a significantly reduced LVEDD (−6.05 mm) and improved prognosis on the basis of optimized medical treatments for CHF [27].

Previous studies have suggested that NPY is a potential regulator of energy homeostasis, but whether it is an important regulator of metabolic remodeling during the acute phase of AAC-induced cardiac pressure overload has not been determined. In this study, we found that serum NPY was immediately induced by 2 days after AAC, as was myocardial expression of the NPY receptors, suggesting that the NPY system may be involved in the process of early energy disturbance of the myocardium. Previous studies in patients with end stage renal disease indicated that elevated NPY is independently associated with LV concentric hypertrophy and systolic dysfunction [14]. Moreover, an NPY-Y2R polymorphism may interact and influence such an association [28], suggesting a potential role for the NPY system in the pathogenesis of ventricular hypertrophy. Similarly, a study in a rat model of CHF induced by aortocaval fistula indicated that increased NPY and its interactions with NPY receptors are involved in cardiac remodeling [16]. A recent in vitro study found that NPY may damage the integrity of mitochondrial structure and therefore disrupts energy metabolism in cultured cardiomyocytes [29]. The results of the above studies, in combination with ours, suggest that NPY and its receptors in the myocardium may mediate the early metabolic dysfunction in the acute phase of AAC-induced cardiac pressure overload, and TMZ may benefit the myocardium during this process by modulating the NPY system. As for the potential mechanisms underlying the potential regulatory effect of TMZ on the NPY system seen in our study, some previously published studies may provide some evidence. A previous study indicated that TMZ effectively inhibits myocardial fibrosis via attenuation of reactive oxygen species (ROS)-related myocardial injury [20]. Interestingly, ROS have been suggested to be an upstream stimulator of the systematic NPY system in the neural tissue [30, 31]. Therefore, TMZ may regulate the NPY system via its inhibitory effect of ROS. Specifically, the exact molecular mechanisms underlying the role of the NPY system in the pathogenesis of pressure overload-induced acute metabolic remodeling and the potential functions of different NPY receptors warrant further investigation.

Our study has limitations that should be considered when interpreting the results. First, because our study was designed to explore the temporal changes in myocardial metabolism remodeling following pressure-overload induced ventricular hypertrophy and the potential favorable effect of TMZ on myocardial metabolism remodeling, we are most interested in the changes in metabolism-related parameters before significant morphological myocardial hypertrophy. Therefore, we chose 7 days of AAC as the main exposure factor. The changes in myocardial metabolism remodeling and potential effects of TMZ after 7 days of AAC should be evaluated in further studies. Moreover, myocardial ADP/ATP, although an important index of myocardial energy status, was not sufficient to reflect the metabolic status of the myocardium following acute pressure overload. Changes in many other parameters related to myocardial energy metabolism, such as myocardial expression of fatty acid or glucose oxidation genes and insulin signaling genes, should be investigated. Finally, the serum level of NPY may not be necessarily correlated with their levels in myocardium. This is important, because we did observe that although NPY expression in the serum decreases with TMZ treatment, the expression of NPY-Rs in the myocardium actually increases, which in our point of view, may be a feedback response of myocardium to the decreased serum NPY. The results of our study could only provide associations between changes of NPY system and TMZ treatment, and the physiological influence and subsequent signaling pathways of changes of NPY system deserve further investigation.

## Conclusions

In conclusion, our study found that cardiac metabolic remodeling is an early change occurring in the myocardium before the observance of typical morphological ventricular remodeling following cardiac pressure overload, and pretreatment with TMZ may at least partly reverse the acute metabolic disturbance, perhaps via regulation of the NPY system. Further studies are needed to clarify the exact molecular mechanisms and potentially distinct role of NPY receptors during this pathogenesis process.

## Abbreviations

AAC: Abdominal aortic constriction
ANF: Atrial natriuretic factor
CAS: Cell surface area
CHF: Congestive heart failure
HW: Heart weight
IVSd: Interventricular septal thickness at end diastole
LVEDD: Left ventricular end-diastolic dimension
LVEF: Left ventricular ejection fraction
LVESD: Left ventricular end-systolic dimension
LVFS: Left ventricular factional shortening
LVPWd: Left ventricular posterior wall thickness at end diastole
MDA: Malonaldehyde
NPY: Neuropeptide Y
SOD: Superoxide dismutase
TMZ: Trimetazidine
